# Machine Learning-Based Texture Analysis in the Characterization of Cortisol Secreting vs. Non-Secreting Adrenocortical Incidentalomas in CT Scan

**DOI:** 10.3389/fendo.2022.873189

**Published:** 2022-06-17

**Authors:** Roberta Maggio, Filippo Messina, Benedetta D’Arrigo, Giacomo Maccagno, Pina Lardo, Claudia Palmisano, Maurizio Poggi, Salvatore Monti, Iolanda Matarazzo, Andrea Laghi, Giuseppe Pugliese, Antonio Stigliano

**Affiliations:** ^1^ Endocrinology, Department of Clinical and Molecular Medicine, Sant’Andrea University Hospital, Sapienza University of Rome, Rome, Italy; ^2^ Department of Surgical and Medical Sciences and Translational Medicine, Sant’Andrea University Hospital, Sapienza University of Rome, Rome, Italy

**Keywords:** adrenal incidentalomas, differential diagnosis of adrenal mass, subclinical hypercortisolism, cortisol secreting adrenal mass, non-secreting adrenal mass, radiomics, texture analysis

## Abstract

New radioimaging techniques, exploiting the quantitative variables of imaging, permit to identify an hypothetical pathological tissue. We have applied this potential in a series of 72 adrenal incidentalomas (AIs) followed at our center, subdivided in functioning and non-functioning using laboratory findings. Each AI was studied in the preliminary non-contrast phase with a specific software (Mazda), surrounding a region of interest within each lesion. A total of 314 features were extrapolated. Mean and standard deviations of features were obtained and the difference in means between the two groups was statistically analyzed. Receiver Operating Characteristic (ROC) curves were used to identify an optimal cutoff for each variable and a prediction model was constructed *via* multivariate logistic regression with backward and stepwise selection. A 11-variable prediction model was constructed, and a ROC curve was used to differentiate patients with high probability of functioning AI. Using a threshold value of >−275.147, we obtained a sensitivity of 93.75% and a specificity of 100% in diagnosing functioning AI. On the basis of these results, computed tomography (CT) texture analysis appears a promising tool in the diagnostic definition of AIs.

## Introduction

Adrenal nodular disease is a frequently increasing in the general population with an incidence that reaches almost 10% in the seventh decade of life (1). More and more pieces of evidence show these lesions discovered through diagnostic imaging [CT and Magnetic Resonance (MR)] performed for other medical problems. This condition characterizes the definition of AI, which, since the early 1990s, has opened up new scenarios regarding their impact on endocrine-metabolic disease (2). Therefore, on the basis of their functional activity, the adrenal nodules have been classified as non-secreting, characterized by an overt hormonal secretion and with subclinical cortisol secretion. According to criteria currently in use, functional characterization of an AI provides a biochemical study through 1-mg overnight dexamethasone suppression test (DST) and dosage of cortisol the following morning (3). On the basis of these indications, patients who suppressed their cortisol at levels above 1.8 mcg/dl (50 nmol/L) were classified as affected by subclinical hypercortisolism. Over the years, this definition has undergone revisions up to the definition of “possible autonomous cortisol secretion” (PACS) with the cortisol results greater than 50 nmol/L and up to 138 nmol/L on DST and of “autonomous cortisol secretion” (ACS) when the cortisol exceeds 138 nmol/L at the DST according to the current nomenclature of the European Society of Endocrinology (4). Several problems interfere in the correct characterization of functional status, such as age, intestinal absorption, and, more frequently, drugs assumption. In fact, overall in elderly patients, most of them are need to take many medications, potentially involved in dexamethasone metabolism.

Radioimaging is able to discriminate the nature of masses through the Hounsfield unit (HU) determination performed by CT scan or chemical shift by MR but cannot predict the hormonal status. Certainly, overt cortisol secretion is associated by many metabolic comorbidities (5). Along this certain acquisitions, many data in the literature have associated PACS and more ACS with different metabolic issues, such as impaired fasting glucose (IGF), impaired glucose tolerance (IGT), overt diabetes mellitus, hypertension, dyslipidemia, and osteoporosis, too (4). This emphasizes the importance of optimizing the diagnosis. Machine learning concept, through different algorithms, offers the possibility to study several biological processes, obtaining quantitative information from imaging and correlating it with outcomes. Radiomics is an emerging technique that translates radiological images into quantitative data to yield biological information and permits an in depth radiological characterization, thus improving diagnosis, decision support, and follow-up monitoring. It is a multistage process in which features based on shape, pixel densities, and texture are extracted from CT or MR images (6).

This methodology, overcoming the qualitative imaging by turning it into valuable predictive outcomes, offers indisputable advantages on the same CT scan images performed to make the diagnosis. In addition, as in this case, it could permit to obtain some information not easy to find.

Very recently, several papers appeared regarding texture analyses in patients with adrenal masses. Specifically, some studies have investigated the ability of texture analysis to improve the diagnosis of adrenal masses. These demonstrated that this technique has a potential role in distinguishing malignant from benign primary adrenal lesions (7–9). Another retrospective study showed how the texture allowed to achieve accurately differentiation of metastatic and benign adrenal masses (10). Starting from the assumption of the extreme intra-tumoral heterogeneity of solid lesions and the ability of various methods used for the interpretation of the tumor texture, an interesting paper proposes a new method based on tree-based analysis of adrenal lesions that provides encouraging results in the characterization of malignant from benign and functioning from non-functioning adrenal masses (11).

On this basis, we use radiomics on two groups of patients with not secreting and cortisol secreting AIs.

## Materials and Methods

### Patients

A retrospective study was performed recruiting 72 patients of both sexes of age ranging from 36 to 84 years, with single adrenal lesion of >1 cm in dimension. Patients belonging adrenal outpatients of Sant’Andrea Hospital Sapienza University of Rome. They discovered incidentally an adrenal mass during radiological examination performed for any clinical problems. Patients were studied following the European Society of Endocrinology/European Network for the Study of Adrenal Tumours (ESE/ENSAT) current criteria practice guideline (4) to exclude any hormonal hypersecretion considering in this study only not secreting and cortisol secreting adrenal masses by DST.

The study was conducted according to the guidelines of the Declaration of Helsinki. Written informed consent was obtained from all participants included in the study. Patient’s inclusion criteria were as follows: (i) adrenal mass of >1 cm incidentally discovered with a CT scan, (ii) cortisol suppressed with DST (non-secreting group), and (iii) failure to suppress cortisol with dexamethasone (cortisol secreting group). The exclusion criteria were as follows: (i) AIs of <1 cm in maximum diameter, (ii) multiple mono- or bilateral adrenal lesions, (iii) patients taking drug potentially interfering with dexamethasone metabolism, (iv) clinical condition affecting cortisol assay, and (v) absence other adrenal hormones hypersecretion (mineraloactive, androgen, and catecholamines). Patients were studied with DST at 11.00 pm. Serum cortisol were collected at 8:00 AM the day after test and determined by the RIA kit (ALPCO, Salem, NH, USA).

On the basis of cortisol value, they were divided in two groups: functioning (32) and non-functioning (40) AIs with cortisol values of >50 and <50 nmol/L, respectively.

### CT Imaging and Radiomic Analysis

All patients underwent an abdominal CT scanner performed on a 128-slice CT scanner (GE Revolution) using a protocol including a preliminary non-enhanced phase and a contrast enhanced study based on an arterial, venous, and delayed phase. Contrast enhancement was performed using IV administration of 110–120 ml of the nonionic contrast medium iomeprolo (350 mg I/mL, Iomeron^®^, Bracco) during arterial, portal venous, and delayed phases and determined using bolus tracking (respectively, 15 s from the peak, 45 min from the arterial phase, and 15 min from the injection. The complete detailed acquisition protocol is included in the **Supplementary Materials**. Preliminary non-contrast phase (2.5 mm thickness) was selected for radiomic analysis.

**Figure 1:**
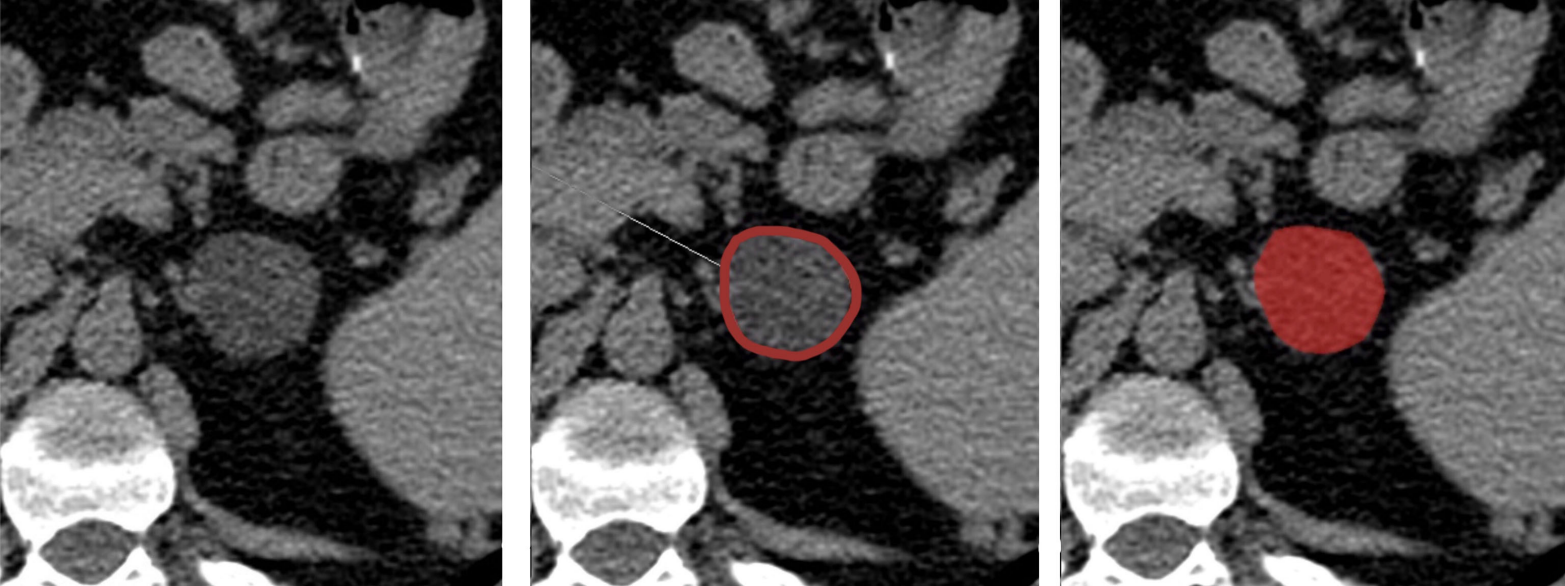
CT ROI segmentation within left adrenal lesion.

All DICOM images were transformed into Bitmap (BMP) format, and the texture analysis was performed using a dedicated software (MaZda statistical texture analysis software, version 4.6.2, available at http://www.eletel.p.lodz.pl/programy/mazda/). The slice containing the maximum diameter of the lesion was selected and an experienced radiologist manually placed a region of interest (ROI) within the boundary of the mass on the selected slice for each patient (**Figure 1**). In this part of the analysis, the radiologist was blinded to the patient condition, not knowing which were the functioning and non-functioning AIs.

To reduce the influence of contrast and brightness variations that could affect radiomic feature quantification, a gray-level normalization of each ROI was necessary. Therefore, CT images were discretized (256 bins, bin width = 16) and normalized using Mazda implemented method, excluding gray levels outside the range μ ± 3σ, where μ was the mean value of gray levels inside the ROI and σ was the standard deviation (SD) of the gray levels inside the ROI.

The analysis of each ROI included features derived from first-order histogram (HIS), co-occurrence matrix (COM), run-length matrix (RLM), absolute gradient (GRA), autoregressive model (ARM), and discrete Haar wavelet transform (WAV), for a maximum of 291 features, as shown specifically in the **Supplementary Materials**, to which are added 23 features regarding the ROI area. HIS features represent the distribution of density within the segmentation area. COM features depends on pairs of pixels/voxels and provide information on lesion heterogeneity. RLM parameters are calculated for four directions, reporting the number of times a run of pixels with a certain gray level is present. ARM and WAV, the high-order features, are usually centered on matrices that examine relationships between three or more pixels*;* specifically, ARM is based on local interaction between pixels in which the density of each pixel is assumed to be the weighted sum of neighboring densities (12–14). At the end of the process, 314 features in total were extracted and analyzed. Post-processing, including lesion segmentation and radiomic analysis, took approximately 3 to 5 min per lesion.

### Statistical Analysis

The 314 extracted features were divided into two groups for the statistical analysis: one group made of constant features (302), namely, the ones extracted *via* texture analysis from every analyzed lesion, and one group of inconstant features (12), composed of the ones extracted by the software only in some of the lesions and not in every one of them.

The statistical analysis was performed using MedCalc (MedCalc statistical software, version 15.8).

Mean and SDs of extrapolated features were obtained and the difference in means between the two groups was statistically analyzed by a two-tailed Mann–Whitney test. Moreover, we enacted a feature robustness analysis to repeated segmentations. Specifically, we obtained two ROI sets, traced by the same operator, for 30 lesions chosen randomly among the two groups. Then, an intraclass correlation coefficient (ICC) analysis was performed for each one of the 302 constant features.

ROC analysis and AUC calculation were used to evaluate the diagnostic accuracy of each feature.

By using only the constant features, a prediction model was constructed *via* multivariate logistic regression with backward and stepwise selection and bootstrap validation.

**Figure 2:**
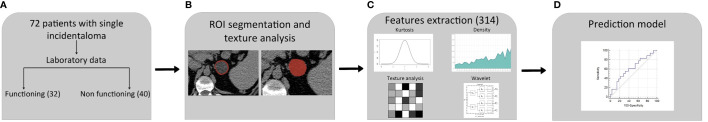
Radiomics workflow and predictive model setup procedure. **(A)** Patients included in the study: 18 of them showed a cortisol secreting and 28 non-functioning adrenal masses. **(B)** Definition of ROI. **(C)** Extraction of 314 features candidates. **(D)** Construction of predictive model by a multivariate logistic regression with eight different variables.

An optimal cutoff was obtain by performing a ROC analysis on the results of the prediction model (**Figure 2**).

## Results

### Hormonal Status and Patients Features

DST identified two series of patients affected by AI with different hormonal secretion. We found 56% (40 patients) affected by not functioning adrenal mass and 44% (32 patients) with cortisol secretion. The mean ± SD age of the secreting group was 65.0 ± 10; the mean ± SD age of non-secreting group was 62.2 ± 11.6. **Table 1** shows patients and adrenal masses features. Mean cortisol values after DST were 95.7 ± 49.7 and 34.4 ± 8.6 nmol/L (p < 0.0001) for secreting and non-secreting lesions, respectively. Size maximum diameter was 28.7 ± 10.2 mm in cortisol secreting and 23.7 ± 7.6 mm in non-secreting AIs (p 0.0188) (**Table 1**).

**Table 1:** Patients and adrenal mass features.

Groups	Secreting Masses	Non-Secreting Masses	P-value
**Number**	32	40	
**Sex**	F = 24	F = 28	NS
	M = 8	M = 12	
**Age**	65 ± 10	62.2 ± 11.6	NS
**DST mean value**	95.7 ± 49.7	34.4 ± 8.6	<0.0001
	(50.0–270	13.9–48.3	
**Size mass**	28.7 ± 10.2	23.7 ± 7.6	0.0188

NS, not significant.

### Texture Analysis

In the group of the 302 constant features, 24 of them showed a different statistically significant distribution (p < 0.05) among the two groups. The feature robustness analysis showed an ICC >0.85 for all the 302 features and an ICC >0.99 for 269 among them.

Using ROC analysis and AUC calculation, 25 constant features showed a p value < 0.05, with an accuracy of 69.44% (**Table 2**).

**Table 2:** Different distribution and ROC analysis of constant features.

Constant Features	Different Distribution (Mann–Whitney Test)		ROC Analysis			
	P-value	AUC	P-value	Sensitivity	Specificity	Accuracy
AreaGr	0.018	0.716	0.0007	56.25%	85%	69.44%
Horzl_GLevNonU	0.0322	0.648	0.0275	59.38%	72.50%	62.50%
S(4,4)SumOfSqs	0.0513	0.634	0.0438	75%	52.50%	58.33%

However, it is still worth saying that, among them, 23 were extrapolated by the data concerning the area of the lesion and that they showed comparable values of sensitivity, specificity, and accuracy.

Among the inconstant group (12), only one (WavEnHL_s-8) showed a different statistically significant distribution (p < 0.05) among the two groups.

ROC analysis of the same feature showed both a sensitivity and specificity of 100%. However, it is important to point out that it was extrapolated only in 10 lesions (seven functioning and three non-functioning) out of the 72 in total.

### Construction of Predictive Model

A predictive model composed of 11 variables was constructed by using a multivariate logistic regression with backward and stepwise selection with the following constant features: AreaGr (Absolute Gradient Area), Perc 01%, S(0,1) Correlat, S(2,-2) SumEntrp, S(3,−3) Contrast, S(4,0) SumEntrp, S(4,4) SumOfSqs, S(4,−4) Entropy, S(5,5) SumAverg, 135dr_RLNonUni, and WavEnLL_s-2. Every one of the 11 variables showed an ICC >0.992.

**Figure 3:**
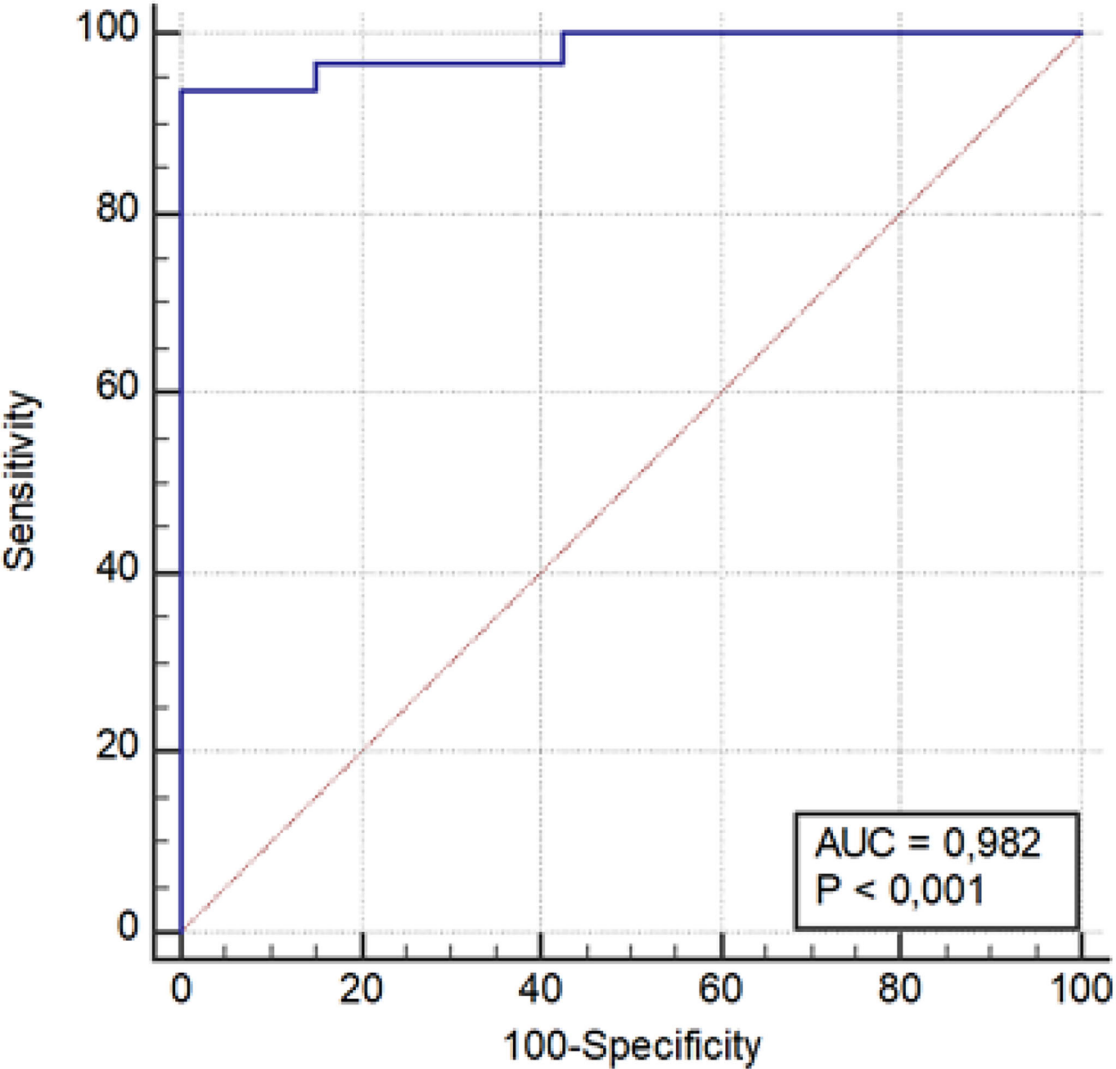
ROC curve graph of eight-variable predictive model.

A score point was assigned to each patient based on the variables composing the predictive model. Specifically, the total score point for each patient was obtained from the sum of the products of each variable’s value, multiple for its coefficient obtained using the multivariate logistic regression. A ROC curve was used to differentiate patients with high probability of functioning masses (AUC 0.982; P < 0.0001). Regarding the score point, using a threshold value >−275.147, we obtained a sensitivity of 93.5% and a specificity of 100% (PPV, 100%; NPV, 99.4%) in diagnosing functioning AI (**Table 3** and **Figure 3**).

**Table 3:** Eleven-variable predictive model: values and coordinates of the ROC curve analysis.

Predictive Model ROC Analysis (11 Variables)	
AUC	0.982
P-value	<0.0001
Threshold value	>−275.147
Sensitivity	93.50%
Specificity	100%
PPV	100%
NPV	99.40%

## Discussion

The evidence of adrenal masses is increasingly frequent in the general population, especially in the elderly. Their discovery primarily raises the problems of diagnostic interpretation relating to the nature of the lesion and the hormonal activity related to them (4). Radiological methods traditionally used allow to discriminate with a sufficient sensitivity and specificity a malignant lesion from a benign one. In fact, through the HU measurement in the basal and post-contrast sequences, CT is capable of discriminating a lesion. Similarly, the chemical shift method in MR allows to obtain precise information on the adrenal masses (15, 16). However, both of these methods lack sufficient diagnostic specificity and sensitivity in more than 30% of AIs. These gaps, relating to the histopathology of the adrenal masses, have recently been addressed by two interesting studies performed with radiomic analysis (7, 9). It allowed to differentiate radiologically dubious benign adrenal masses from adrenal cortical carcinomas, proposing as a useful tool in clinical decision-making. Biochemical activity of AIs is commonly investigated by the most widespread methods such as Radio ImmunoAssays (RIA) and Enzyme-Linked Immuno Assay (ELISA) in clinical practice, and, more recently, high-performance liquid chromatography–mass spectrometry (HPLC-MS) is an advanced technological tool for the characterization of these lesions. However, although these represent the diagnostic cornerstones of AIs, there are numerous conditions that make it difficult to correctly establish hormonal activity of an adrenal mass. These can be essentially traced back to extrinsic and intrinsic causes. Among the former, many substances and drugs notoriously interfere on hypothalamus–pituitary–adrenal axis (HPA). First of all, exogenous glucocorticoids with a strong inhibitory effect followed by many others (opioids, serotonin antagonists, tricyclic anti-depressants, dopamine agonists, antipsychotics, and so on) (17). Vice versa, serotonin agonists and dopamine antagonists have a stimulatory effect on the HPA axis (18). Notoriously, the frequency of adrenal masses is more frequent in the elderly (4), when many of these drugs are prescribed for other diseases. Intrinsic causes of HPA axis control arise from fine physiological mechanisms. In fact, cortisol secretion is modulated by several classes of hormones such as estrogen, GH, desmopressin, thyroid hormones, and cytokines, but also lifestyle habits such as sleep and wakefulness have a non-negligible role (19). Finally, many abusive substances such as nicotine and alcohol are known to perturb HPA axis (20, 21). Often, these obstacles are the cause of a delayed or omitted diagnosis of hypercortisolism.

Radiomics provides quantitative and reproducible information easily deducible from different radiological and medical nuclear sources, thus allowing its use in multicentric longitudinal observational studies (22). It allows, by analyzing the density of the tissue, their shape, and other parameters, to extrapolate, through mathematical algorithms, information in that particular setting. Furthermore, this whole process does not affect the CT exam in terms of time and radiation dose. In fact, it is included in the post-processing and only takes a few minutes.

Recently, several papers have been published regarding to the application of radiomics in patients with adrenal lesions. Results coming from these studies, performed especially to discriminate benign from malignant lesions, appear to be very promising (23). Keeping in mind that approximately 50% of AIs produce more cortisol and about diagnostic pitfalls occurring in adrenal masses characterization, we speculate on the properties of radiomics to discriminate cortisol-secreting from not-secreting AIs.

In particular, we compared two series of patients with cortisol secreting and non-functioning adrenal mass, respectively. We have chosen patients with certain hormonal secretion stable with at least three times with DST in the 3 years preceding the study and with unchanged mass size over time. Choosing to ascertain such stability over time proved to be a really narrow inclusion criteria, reducing greatly the final number of patients in the study. However, having the certain of functionality and non-functionality of the AIs allowed us to produce an even more significative prediction model.

Patients with mineral corticoids, sex hormone, and cathecolamines excess were excluded from the present study. DST clearly placed patients in two defined groups: patients with non-secreting nodules had a cortisol level of 34.4 ± 8.6 nmol/L, those with secreting nodules had a cortisol level of 97.1 ± 49.9 nmol/L (p < 0.0001) (**Table 1**).

In this study, more than 300 features from texture analysis have been considered in all 72 patients studied (32 cortisol-secreting and 40 non–hormone-secreting AIs).

Depending on their presence in every lesion or just in some of them, the features were divided in constant (302) and inconstant (12).

Interestingly, regarding 24 constant features and one inconstant feature, this comparison revealed a statistically significant difference among the two groups (p < 0.05). Furthermore, this single inconstant feature (WavEnHL_s-8) present in just 10 lesions (seven functioning and three non-functioning) showed both a sensitivity and specificity of 100%. The construction of a model based on a multivariate logistic regression with backward and stepwise selection of eleven constant features [AreaGr, Perc 01%, S(0,1) Correlat, S(2,−2)SumEntrp, S(3,−3)Contrast, S(4,0)SumEntrp, S(4,4)SumOfSqs, S(4,−4)Entropy, S(5,5) SumAverg, 135dr_RLNonUni, and WavEnLL_s-2] allowed us to obtain a predictive model to discriminate the functional characteristics of an adrenal mass. The ROC curve, shown in **Figure 3**, indicates the ability of this method to differentiate cortisol secreting AIs (AUC 0.982; P < 0.0001) (**Table 3**). Using a threshold value >−275.147, we obtained a sensitivity of 93.5% and a specificity of 100% in diagnosing functioning adrenal mass (PPV 100%; NPV 99.4%) (**Table 3**).

On the basis of this predictive model, the ones with a threshold value >−275.147 have a high probability of being functioning and vice versa the ones with a value <−275.147 have a high probability of being non-functioning. In particular, machine learning applied to radiological techniques seems to discriminate, through the data that we demonstrate here, the functional activity of an adrenal mass. Shoemaker et al. exploring, in a retrospective series, the ability of radiomics to discriminate some important parameters within adrenal lesions, provide encouraging results: malignant from benign (AUC 0.78), and functioning from non-functioning (AUC 0.93) and calcified from non-calcified (AUC 1) (11). According to these results about hormonal activity of adrenal masses, we are able to confirm, improving, the AUC with 0.98 in our series of selected AIs (**Figure 3**). Another study proposed radiomics to distinguish cortisol-secreting from aldosterone-secreting adenoma in an integrating model using the combination of radiomic signature and clinic-radiological features. The authors found a good sensitivity and specificity in this integrated model for distinguish hormone-secreting functional adrenal adenomas subtypes (24). All together, these studies confirm the ability of radiomics to address a hormonal secretion.

However, we are aware that this study suffers of some limitations, mainly represented by a retrospective analysis on a poor series of AIs caused by the narrow inclusion criteria. Because of that, to reduce the bias occurring, we adopt more stringent criteria. First, we included only those patients that showed a consistent hormonal profile during the 3 years, excluding discontinuous hormonal secretion. Second, we considered only adrenal mass higher than 1 cm to discriminate the correct masses’ border without any increase in mass size. Third, we excluded multiple monolateral and bilateral adrenal masses occurring during the follow-up. Again, we excluded also patients taking drug potentially involving in dexamethasone metabolism and those with clinical condition affecting cortisol assay being the majority of patients enrolled in this study in the middle and advanced age range (50–70 years). In addition, even radiomics, like any technique, has some limits, including susceptibility toward image acquisition and reconstruction parameters and pitfalls that can lead misinterpreting data.

One of the limits of our study is the high number of features included in the predictive model. We are aware that this could be associated with a relative risk of overfitting based on the obtained results; in this context, further perspective studies are necessary to validate this model, also based on external data and different CT scanner to value its generalizability. Furthermore, we care to emphasize that this is still a pilot study and more investigations need to explore this method in a particular setting obtaining reliable data.

Up to now, most of radiomics studies have been performed in oncologic field with surprising results (7). Furthermore, as supported by the review from Crimì et al. (23), encouraging data come from texture analysis in adrenal lesions. This study moves the field of application of radiomics in the context of functional adrenal disease trying to overcome some of the pitfalls described above, proposing it as a useful tool for clinical investigation. Suitably, Zheng et al. suggest to extend radiomics to the study of secreting and non-secreting adrenal adenomas (24). Moreover, a clinical validation of a predictive model can represent a valid tool to discriminate the hormonal source occurring in bilateral adrenal lesions (micro- and macronodular adrenal disease). Therapeutic goals of these applications would be represented by a proper indication to surgical procedure for all those overt hormonal conditions including Conn’s syndrome and bilateral macronodular adrenal hyperplasia (BMAH).

Data resulting from this paper are the result of a very preliminary study worthy of being validated in a large prospective multicentric studies.

Furthermore, a latest review of the current literature proposed a Radiomics Quality Score (RQS) to assess the quality of published results (25). Very recently, another review analyzed several studies on the application of radiomics in the field of adrenal disease, using RQS to assess their methodological quality (26). In this perspective, we applied this concept to our study and obtained an RQS of 7 (higher so far than those calculated for evaluation of the adrenal masses).

In conclusion, we have preliminarily investigated a machine learning-based texture analysis performance in the characterization of cortisol from non-secreting AIs in CT scan. This method may provide an additional opportunity in improving diagnosis of AIs whenever biochemical tests are found to be inapplicable or inconsistent.

## Data Availability Statement

The raw data supporting the conclusions of this article will be made available by the authors, without undue reservation.

## Ethics Statement

Ethical review and approval was not required for the study on human participants in accordance with the local legislation and institutional requirements. The patients/participants provided their written informed consent to participate in this study.

## Author Contributions

IM and AS conceived the original idea. RM, PL, MP, and SM enrolled the patients and performed the hormonal study. FM, GM, CP, and BD performed radiological imaging. FM and GM performed radiomics analysis. FM, BD, and AL validated radiomics methods. RM, FM, IM, and AS wrote the manuscript with support of BD. AL, GP, and AS supervised the project. RM and AS performed data curation. AS acquired funding. All authors contributed to the article and approved the submitted version.

## Funding

This work is supported by Sapienza Progetto d'Ateneo n. RM12117A882208F6. The funding source had no role in the design of the study or in the analysis and interpretation of results.

## Conflict of Interest

The authors declare that the research was conducted in the absence of any commercial or financial relationships that could be construed as a potential conflict of interest.

## Publisher’s Note

All claims expressed in this article are solely those of the authors and do not necessarily represent those of their affiliated organizations, or those of the publisher, the editors and the reviewers. Any product that may be evaluated in this article, or claim that may be made by its manufacturer, is not guaranteed or endorsed by the publisher.
