# Supplementary figures and images for: Machine Learning-Based Texture Analysis in the Characterization of Cortisol Secreting vs. Non-Secreting Adrenocortical Incidentalomas in CT Scan
Source: Front Endocrinol (Lausanne). 2022 Jun 17;13:873189. doi: 10.3389/fendo.2022.873189 (PMC9248203; doi:10.3389/fendo.2022.873189)

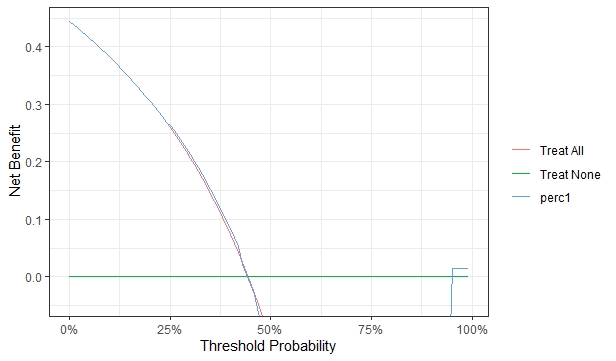

Supplement: Supplementary file 1 [file Image_1.jpeg]
