# Supplementary material for: Machine Learning-Based Texture Analysis in the Characterization of Cortisol Secreting vs. Non-Secreting Adrenocortical Incidentalomas in CT Scan
Source: Front Endocrinol (Lausanne). 2022 Jun 17;13:873189. doi: 10.3389/fendo.2022.873189 (PMC9248203; doi:10.3389/fendo.2022.873189)
Supplement: Supplementary file 2 [file DataSheet_1.docx]

**CT protocol**

| **Parameters** | **Value** |
| --- | --- |
| Tube voltage | 120 kVp |
| Tube current | 150-400 mA |
| Effective section thickness | 2.5 mm |
| Reconstruction interval | 1.25 mm |
| Gantry rotation time | 0.4 s |
| FOV (Field Of View) | 350 x 350 mm |
| Reconstruction matrix | 512 x 512 |

All patients underwent an abdominal CT scanner performed on a 128 slice CT scanner using a protocol including a preliminar non enhanced phase and a contrast enhanced study based on an arterial, venous and delayed phase.

Contrast enhancement was performed using IV administration of 110-120 mL of the nonionic contrast medium iomeprolo (350 mg I/mL, Iomeron^®^, Bracco) during the arterial, portal venous and delayed phases, as determined using bolus tracking (respectively 15 sec from the peak , 45 min from the arterial phase and 15 min from the injection).

**Mazda’s texture features**

Totale number of texture features: 279-291

1. **HIS (first-order histogram)**

- Mean (histogram’s mean)
- Variance (histogram’s variance)
- Skewness (histogram’s skewness)
- Kurtosis (histogram’s kurtosis)
- Perc.01% (1% percentile)
- Perc.10% (10% percentile)
- Perc.50% (50% percentile)
- Perc.90% (90% percentile)
- Perc.99% (99% percentile

Total number of histogram based features: 9

1. **GRA (absolute gradient)**
2. GrMean (absolute gradient mean)
3. GrVariance (absolute gradient variance)
4. GrSkewness (absolute gradient skewness)
5. GrKurtosis (absolute gradient kurtosis)
6. GrNonZeros (percentage of pixels with nonzero gradient)

Total number of absolute gradient based features: 5

1. **RLM (run-length matrix)**

- RLNonUni (run length nonuniformity)
- GLevNonU (grey level nonuniformity)
- LngREmph (long run emphasis)
- ShrtREmp (short run emphasis)
- Fraction (fraction of image in runs)
- Features are computed for 4 various directions.

Total number of run length matrix based features: 20

1. **COM (co-occurrence matrix)**

- AngScMom (angular second moment)
- Contrast (contrast)
- Correlat (correlation)
- SumOfSqs (sum of squares)
- InvDfMom (inverse difference moment)
- SumAverg (sum average)
- SumVarnc (sum variance)
- SumEntrp (sum entropy)
- Entropy (entropy)
- DifVarnc (difference variance)
- DifEntrp (difference entropy)

Features are computed for 5 between-pixels distances (1, 2, 3, 4, 5) and for 4 various directions.

Total number of co-occurrence matrix based features: 220

1. **ARM (autoregressive model)**

- Teta1 (θ1)
- Teta2 (θ2)
- Teta3 (θ3)
- Teta4 (θ4)
- Sigma (σ)

Total number of autoregressive model based features: 5

1. **WAV (Haar wavelet transform)**

- WavEn (wavelet energy)

Feature is computed at 5-8 scales within four frequency bands LL, LH, HL and HH.

Total number of Haar wavelet-based features: 20-32
